# Inhalation-Pneumonia

**Published:** 1897-10

**Authors:** W. L. Williams, P. A. Fish

**Affiliations:** Professor of Surgery in the New York State Veterinary College; Professor of Therapeutics in the New York State Veterinary College


### INHALATION-PNEUMONIA.

By W. L. Williams, V.S.,
PROFESSOR OF SURGERY IN THE NEW YORK STATE VETERINARY COLLEGE;

AND

P. A. Fish, D.V.S.,
PROFESSOR OF THERAPEUTICS IN THE NEW YORK STATE VETERINARY COLLEGE.

The inhalation of foreign bodies, mechanical, chemical, or bacterial, tends usually toward bacterial invasion of the bronchial mucosæ, extending thence to the deeper parts, finally involving all tissues of the lungs, inducing suppuration, necrosis, and death.

The symptoms vary greatly in detail, though in general they present the ordinary signs of bronchitis and pneumonia, along with expectoration of fetid bronchial secretions and variations in chest-sounds as would result from the presence in the tubes of the foreign bodies inhaled or of the products of disease.

The most common causes are the inhalation of medicines during their forced administration; of food-particles during coma, as in parturient apoplexy of the cow; of pathogenic organisms and their products, after arytenectomy for the cure of laryngismus in horses, or other operations in the upper air-passages; by the inhalation of pus discharged into the fauces or upper air-passages, from abscesses, diseased teeth, or tumors; by animal parasites in the air-passages; by the inhalation of irritant gases or hot smoke or of liquid chloroform during the production of anæsthesia, and by a great variety of more rare accidents ending in the lodgement of irritant foreign bodies within the air-passages. We might include, also, a highly important class of infections, like diphtheria, in which there is a tendency to the extension of the lesions to the lungs; or of tuberculosis, actinomycosis, and glanders, in which necrosis and softening of patches of lung-tissue frequently occur, discharging into the bronchi, tending to pass upward, only to be, in part, carried backward into neighboring bronchi, establishing there their typical pathological processes in the manner commonly termed auto-infection.

As a rule, the treatment of these cases has proved ineffectual, and practitioners recoil from them with well-founded dread. The plan usually followed consists in the internal administration of expectorants and sedatives, with some of the gum-resins possessing antiseptic properties, and which are largely excreted by the lungs, and the inhalation of vapors, either simple or medicated. Intratracheal injections of vermicides have been successfully employed in verminous bronchitis, and the bronchial mucosa has been used as a prompt and reliable absorbent surface for the administration of various drugs in solutions of small volume. The senior writer has attempted the administration of antiseptics in small volumes by intratracheal injection in cases of suppurative bronchitis, and has endeavored to aspirate suppurative areas of the lung and inject the cavities with antiseptics, but without noteworthy success. Beaumont Small (*Handbook of Medical Science*, ix. 756) employed a 1 : 500 solution of pyoctanin in the form of intrapulmonary injection of eight to sixteen minims in pulmonary tuberculosis, which was reported well-borne, except that when reaching the bronchi it caused violent coughing, but was said to have lessened the hectic condition and diminished the number of bacilli in the sputa.

We have been unable to find a record of any attempts to administer, per trachea, for therapeutic purposes large volumes of liquid, either as mechanical detergents or as topical or general antiseptics, the filling of the lower air-passages with liquids being associated in the popular mind with drowning. Opposed to this fear existed the well-known fact that in partial drowning the water which had well filled the air-passages was in many cases partly drained out, largely absorbed, and the patient left little worse for the experience beyond the psychical shock. It had also been shown experimentally that large quantities of water could be slowly introduced into the lungs through the trachea and become absorbed without untoward results, while a like volume introduced rapidly and persistently would produce profound disturbance and eventually death. Notwithstanding that absorption occurs more rapidly in the lungs than elsewhere in the body, excess of fluid effects material changes not only in the respiratory epithelium, but also in the blood, in which any change must necessarily affect all other tissues.

In an experiment at Lyons, France, under the direction of Gohier, 30 litres ($7\frac{1}{2}$ gallons) of water were injected into the trachea of a horse without causing death. In another case it required 40 litres (10 gallons) to kill the animal by suffocation. Colin (1873, vol. ii. page 109), experimenting along the same line, introduced into the trachea of a horse by means of a special apparatus 6 litres of water per hour at a temperature of 30° to 35° C., which was continued for three and one-half hours, making a total of 20 litres, after which the animal was immediately destroyed, the bronchi quickly opened and found empty, all the water having been absorbed. In another horse he introduced into the trachea 25 litres of water in six hours, and bled him three times at intervals of two hours, obtaining 6 kilogrammes ($13\frac{2}{10}$ pounds) of blood. The respiratory mucosa absorbed all the water without apparent inconvenience to the animal.

Intratracheal medication, though not in general use, has much to recommend it when rapid effects are desired, especially in those pulmonary diseases where antiseptics are indicated. Among the agents best adapted for this use is hydrogen peroxide, which is antiseptic, non-toxic, deodorant, styptic, and, in dilute solution, non-irritant.

With these facts and suggestions before us, two cases were presented at the clinics of the New York State Veterinary College which served to invite more radical attempts at intratracheal medication than had previously to our knowledge been undertaken, the results of which were, to us, at once so unexpected and instructive that we felt ourselves warranted in communicating them to the profession, though admitting that our experiments were preliminary and quite incomplete.

CASE I.—An adult roadster gelding, vigorous and sound so far as known, except well-marked laryngismus paralyticus, on which account he entered the clinic for the removal of the left arytenoid cartilage. After careful dieting, he was cast for the operation on June 3d. General anæsthesia was omitted, and cocaine used to produce local insensibility. A tracheotomy-tube was inserted some twelve inches downward from the larynx, after which the arytenoid cartilage was excised in the ordinary manner by the senior author of this paper. The patient fought viciously throughout the operation, and, the day being warm, he became very hot and bathed in profuse perspiration. The operation completed, the tampon tracheatube was inserted and the operation-field tamponed with absorbent cotton and iodoform.

*June 4th.* The tampon and canula were removed, the operation-field carefully sponged with 1 : 1000 sublimate solution, and the horse was permitted to drink a goodly quantity of milk, which he apparently relished. From this time until June 10th the patient seemed bright, drank liquid food with avidity, temperature was normal, and all appeared well except an abundant and ever-increasing fetid, purulent discharge from the nostrils and tracheal openings.

10*th.* He appeared weaker and had fallen down, but was quickly assisted to his feet, and, the fetor of tracheal discharges still increasing, we injected small quantities of hydrogen peroxide into the trachea, which caused the discharge of some froth.

11*th.* Well-defined suppurative broncho-pneumonia was noted, the patient was rapidly failing, and the area of disease was so great that the intratracheal injection of small volumes of antiseptics could promise no benefit. At this juncture Prof. Law suggested that, as an experiment on a hopeless case, we might, in light of the experiments noted above, attempt the administration of antiseptics by the intratracheal injection of large volumes of liquids and permit them to be absorbed from the pulmonary mucosa. We prepared a tepid solution consisting of 5 litres of water, 30 grammes sodium chloride, and 60 c.c. of the commercial solution of hydrogen peroxide. Placing this in an irrigating reservoir at an elevation of ten feet above the animal, with the liquid gravitating downward through a three-eighth inch rubber-tubing and escaping through a one-fourth inch nozzle, the latter was inserted in the tracheal opening and the liquid allowed to flow into the trachea in a full stream until about one litre had entered, when by an expulsive effort the greater part was thrown out through the tracheal openings—mouth and nostrils—the liquid emerging frothy and carrying with it fetid discharges. As soon as that which had been thrown into the trachea was well out the process was quickly repeated until within ten minutes the entire five litres of liquid had passed into the trachea, the greater part of it having been thrown out again, carrying with it much putrid material. This was accomplished without apparent distress to the patient, causing only a moderate amount of coughing with each expulsive effort, and leaving him at the conclusion of the ordeal apparently without additional fatigue and with the fetor of his breath greatly diminished, his air-passages clean, and, to all appearances, the local conditions materially improved. The patient died on the following day without our having repeated the treatment, and the autopsy showed extensive necrotic broncho-pneumonia.

The only result gathered from the case was the facility with which large volumes of liquids could be rapidly introduced into the trachea without producing inconvenience to the animal worthy of remark, at the same time thoroughly flushing out the air-passages and measurably deodorizing and disinfecting them.

Case II. was in all material respects like Case I. Operated upon on June 4th of this year in the same manner as Case I., by student H. The patient struggled less violently than Case I., and was less fatigued after the operation. Tampon and tampon-canula applied as in Case I., and removed the following day. Deglutition very imperfect, almost all fluids taken into the pharynx being expelled through the nostrils and tracheal openings.

From June 6th to 12th the loss of power of deglutition continued unabated, and there were no notable changes except that gradually increasing fetid discharges took place from the nostrils and tracheal and laryngeal openings. By June 13th the patient had become exceedingly weak, having been practically without food, either solid or liquid, for nine days. At this stage the tracheotomy-tube, which had been removed on June 6th, was replaced as a precautionary measure, and the patient allowed to eat succulent grass and soft bran and linseed mashes, of which he partook sparingly, much of it dropping out through the laryngeal opening.

By June 17th the breath had become very fetid, and, on the 18th, excessively stinking. An examination of the tracheal wound revealed a necrotic piece of cartilage, which was excised. We then introduced into the trachea 5 litres of tepid water with 30 grammes of sodium chloride and 60 c.c. solution of hydrogen peroxide, which, flowing in rapidly, was largely expelled, thoroughly flushing the air-passages, pharynx, and surgical wound, cleansing and deodorizing the parts.

On the 19th the fetor seemed so much less that the irrigation was omitted; but on the 20th the fetor had increased, and the lungs were again flushed out as on the 18th, without inducing any marked discomfort. The intratracheal treatment was now continued.

After this the patient seemed to improve slowly, if at all, in strength, appetite, and power of deglutition, and was greatly harassed by a persistent cough. The tracheotomy-tube was removed on June 25th, as the power of deglutition now seemed restored, and by July 14th the tracheal and laryngeal wounds had closed; but the cough continued, the patient remained emaciated and weak, the appetite indifferent, the breath had again become fetid, especially evident during his fits of coughing, when he expectorated through the mouth or expelled through the nose dirty-gray, very fetid discharges. As there was evidently a still more serious pathological condition present, we reopened the laryngeal and tracheal wounds for examination, finding each completely healed and all adjacent parts apparently normal. We had barely completed our physical examination of the parts when, in a fit of coughing, he expelled through the laryngeal incision an excessively fetid, dirty-grayish, tenacious mass, which it could now be no longer doubted had emanated from low down within the bronchi, and indicated local purulent broncho-pneumonia. We then began anew the irrigation of the bronchi, the volume, composition, and mode of administration of the fluid remaining the same, and being repeated daily. On the 15th we began the internal administration of quinine sulphate ʒj, nux vomica, grs. xx, and arsenic, grs. ij., twice daily. At the first expulsive effort during each irrigation the patient expelled with the water about 10 c.c. of a dirty-gray, very fetid, tenacious discharge, and on July 17th he expelled a piece of fetid necrotic tissue estimated to weigh 2 grammes.

*July 18th.* The volume of water was reduced to 3 litres, the sodium chloride correspondingly, leaving it at .06 per cent., while the volume of hydrogen peroxide was left unchanged.

19*th.* Five days after the beginning of the regular daily irrigations the fetor of the expectorated mass had greatly diminished, while its color had changed to almost that of ordinary mucus.

20*th*. No fetor could be detected in expectorate nor in expired air.

26*th*. The hydrogen peroxide was doubled, which caused more coughing and resulted in increased discharge of bronchial secretion on the 27th and 28th, though the hydrogen peroxide had been reduced on the 27th to the original amount, and was so continued thereafter.

31*st*. The patient had markedly improved in every way, was gaining rapidly in flesh, the cough was less frequent, the bronchial discharge less, and seen practically only at times of irrigation, and the animal would run and play in the paddock. The use of the tracheotomy-tube through which injections were made was dispensed with on July 30th, and the nozzle of the injecting-tube inserted directly in the trachea, with an apparent advantage in causing less coughing.

*August* 2*d*. The patient had so far recovered that treatment was discontinued and the tracheal wound permitted to close.

7*th*. He was hitched to a buggy and tested at a rapid pace up a steep hill, and found apparently much improved in wind.

12*th*. He was driven home, a distance of twenty miles, without showing signs of fatigue. On August 31st the owner reported the patient much improved in flesh, practically free from cough, almost free from respiratory difficulty when driven rapidly, and taking exercise-work daily without fatigue or other difficulty.

While our experiments were very limited in extent, and can be regarded only as preliminary and suggestive, some facts have been established which appear to us of interest.

It has been shown not only that large volumes of water can be introduced slowly into and absorbed from the lungs, but that such quantities can be introduced at a rapid rate into the trachea and bronchi, if the trachea is open, and be thrown back through trachea, larynx, pharynx, mouth, and nostrils, thoroughly flushing these parts, constituting thereby our most efficient cleansing procedure. We have shown that the air-passages tolerate quite well at least one antiseptic—hydrogen peroxide.

Of great interest, it appears to us, is the fact that on July 17th, during irrigation, we flushed out a large-sized piece of necrotic tissue, which must have been lodged low down in the bronchi. In each case apparently, we cleared the bronchi (at least the larger ones) of all foreign matter, and certainly are warranted in believing that the irrigation of the lungs exerted a very favorable influence on the course of the disease.

Our efforts suggest a much wider range of usefulness. In accidental inhalation of drugs during drenching, it seems that irrigation may, in safety, be depended upon to wash out oils, to dilute and wash out such irritants as alcohol, turpentine, whiskey, chloral, etc.; while in case of foreign bodies of considerable size it offers us a means for their removal quite worthy of a trial. It seems quite possible that good results might be had by this plan in such affections as pulmonary tuberculosis, where large softening areas communicate with and discharge into the bronchi, and in all forms of suppurative broncho-pneumonia, and possibly also in extensive diphtheritic invasion of the air-passages.

Perhaps one of its most direct uses will be found in the prevention of inhalation-pneumonia after arytenectomy, as it affords us not only a safe plan for thorough irrigation of the field of operation, but the fluid passing down the trachea into the bronchi will flush out and destroy any pathogenic organisms which have been inhaled.

We do not say that our plan, formula, or rate of administration is the best. Other antiseptics may be better and other rates or details of administration may be far superior. We do not know if it is better to have a tracheal or laryngeal opening or not, though the absence of a counter-opening might, it seems to us, lead to a dangerous spasmodic closure of the larynx. The rate of administration can evidently be varied. We did not know at the beginning of our experiment the rate of administration by the experimenters quoted, and departed widely from their plan by introducing the liquid at a very rapid rate—quite too rapid to permit of total absorption—and in that way learned that we could, without discomfort or injury, have it quickly expelled; and thus we learned by comparison with Colin's and Gohier's experiments that we may, at our option, by varying the rate of administration, either have the liquid absorbed or rejected or partly absorbed and in part expelled. At some times we apparently had 50 per cent. or over absorbed, though always given rather rapidly, while in other cases nearly all appeared to be rejected.

We have been led to hope that, in spite of the meagre experience upon which we have based our communication, the facts and suggestions will suffice to lead others to study the plan of treatment herein outlined, with a view to developing a successful method of therapeutics in this heretofore baffling group of affections.

